# The patient mobility saga continues – Ruling of the Court of Justice of European Union in the case of Elena Petru

**DOI:** 10.3325/cmj.2014.55.441

**Published:** 2014-10

**Authors:** André den Exter

**Affiliations:** Jean Monnet Chair EU Health Law, Erasmus University Rotterdam, the Netherlands

From October 2013, European Union member states are obliged to adopt the new European rules allowing patients to search for health care abroad at the costs of the national authorities. The so-called cross-border care Directive (2011/24/EU) facilitates therefore patient mobility in the European Union (1). But only in case a certain treatment option is not (timely) available, or not equally effective in the country of residence, and when the treatment requested is among the benefits of the home member state. This new regime incorporates previous cross-border care rulings of the European Union’s Court of Justice, which are the main reason of this new Directive. Whereas member states are still in the process of transposing the Directive into national law, a new ruling from this Court has further triggered patient mobility with considerable consequences to low income countries.

## The Petru case (2,3)

Ms Elena Petru had been suffering for some years from a serious cardiovascular disease, for which she underwent a surgical operation. Since her situation deteriorated she was admitted to the Timisoara Institute for Cardiovascular Disease, Romania. After medical examination, the treating physician suggested an open heart surgery in order to replace the mitral valve and insert two stents. In the belief that the hospital’s infrastructure was inadequate for such a procedure, Ms Petru went to a German clinic, where the surgery was carried out. The total costs of the surgery and post-operative hospital expenses were € 17 714. Before going to Germany, Ms Petru required permission from the national health insurance agency for receiving health care abroad, which was denied for the reason that there was no indication since the treatment sought could be provided in Romania within a reasonable time period. Subsequently, Ms Petru lodged a complaint against the national health insurance agency, claiming full compensation for the costs of treatment in Germany.

In a special procedure (“preliminary ruling”), the European Court needed 1 page to decide that Ms Petru should be fully compensated, meaning that the Romanian authorities were responsible for cost reimbursement of the health care services provided, even when the treatment option is available on the Romanian territory. As such, the European Court extended EU citizens’ right to cross-border care which is not without consideration. What is of importance are the circumstances of this particular case. First, the intervention was covered by Romanian health insurance law, therefore considered as an ensured benefit. Second, Ms Petru claimed that due to the lack of medication and basic medical supplies and infrastructure, the treatment could not be provided timely in Romania taken into account her current state of health and the probable course of the disease. According to the Court, such lack of medicines and medical supplies can make it impossible to perform the treatment in good time. As a matter of principle, the Court accepted that argument and therefore ordered Romania to reimburse the health care costs abroad. The outcome might have been different when the same or effectively similar treatment would be available in another Romanian hospital in good time, but that was not the case.

## Individual patient’s victory but….

With this case, the Court confirmed its previous rulings on the patients’ right to receive health care abroad, while taking into account all the relevant circumstances. What is new, is the timely available treatment argument, interpreted as absence of medicines and medical supplies. This interpretation means a further extension of the patient mobility concept in the European Union.

Although one may welcome such a patient friendly outcome, it also raises concerns about the sustainability of particularly low income countries, being confronted with widespread shortages in basic health care resources. Structural shortages of medicines and medical supplies are not uncommon in these countries and therefore justify a more specific approach but the Court was silent about that aspect. Instead, the Court’s top advisor, Advocate General Cruz Villalon suggested to differentiate among occasional and more structural shortages. In case of incidental shortages of supplies, prior authorization for cross-border health care by the home member states cannot be denied, whereas in case of “systemic deficiencies,” ie, structural and prolonged shortages of medicines, medical infrastructure and medical staff, refusal of authorization is allowed since the domestic health financing system cannot afford a mass-exodus of patients in need of medical care. The systemic deficiency scenario limits therefore patient mobility for reasons of public interest, ie, safeguarding the long-term sustainability of the national health care system.

In this particular case, the Court could leave aside the advisor’s opinion since it was not explicitly argued by the Romanian government. It is however a matter of time when the Court will be forced to address the systemic deficiency argument raised by member states in a similar position. So, despite the new cross-border care regime, this patient mobility debate will continue at the European Court.
